# Bifunctional, Copper-Doped, Mesoporous Silica Nanosphere-Modified, Bioceramic Scaffolds for Bone Tumor Therapy

**DOI:** 10.3389/fchem.2020.610232

**Published:** 2020-12-09

**Authors:** Hongshi Ma, Zhenjiang Ma, Qufei Chen, Wentao Li, Xiangfei Liu, Xiaojun Ma, Yuanqing Mao, Han Yang, Hui Ma, Jinwu Wang

**Affiliations:** ^1^Shanghai Key Laboratory of Orthopaedic Implants, Department of Orthopaedic Surgery, Shanghai Ninth People's Hospital, Shanghai Jiao Tong University School of Medicine, Shanghai, China; ^2^Department of Orthopaedic Surgery, Shanghai Zhongye Hospital, Shanghai, China; ^3^Department of Orthopedics, Shanghai General Hospital, Shanghai Jiao Tong University School of Medicine, Shanghai, China

**Keywords:** Cu-containing mesoporous silica nanospheres, 3D printing, bifunctional scaffolds, photothermal tumor therapy, tissue regeneration

## Abstract

In the traditional surgical intervention procedure, residual tumor cells may potentially cause tumor recurrence. In addition, large bone defects caused by surgery are difficult to self-repair. Thus, it is necessary to design a bioactive scaffold that can not only kill residual tumor cells but also promote bone defect regeneration simultaneously. Here, we successfully developed Cu-containing mesoporous silica nanosphere-modified β-tricalcium phosphate (Cu-MSN-TCP) scaffolds, with uniform and dense nanolayers with spherical morphology *via* 3D printing and spin coating. The scaffolds exhibited coating time- and laser power density-dependent photothermal performance, which favored the effective killing of tumor cells under near-infrared laser irradiation. Furthermore, the prepared scaffolds favored the proliferation and attachment of rabbit bone marrow-derived mesenchymal stem cells and stimulated the gene expression of osteogenic markers. Overall, Cu-MSN-TCP scaffolds can be considered for complete eradication of residual bone tumor cells and simultaneous healing of large bone defects, which may provide a novel and effective strategy for bone tumor therapy. In the future, such Cu-MSN-TCP scaffolds may function as carriers of anti-cancer drugs or immune checkpoint inhibitors in chemo-/photothermal or immune-/photothermal therapy of bone tumors, favoring for effective treatment.

## Introduction

The traditional therapy for bone tumors is surgical intervention to remove tumor lesions. However, surgical resection cannot ensure complete excision of all tumor cells, and residual tumor cells may cause tumor recurrence. In addition, large bone defects caused by surgery are difficult to self-repair. Therefore, it is crucial to develop a bioactive biomaterial, whose bifunctionality includes both killing residual tumor cells and promoting bone defect regeneration. Such a biomaterial would also demonstrate great potential for bone tumor therapy.

Mesoporous silica nanospheres (MSNs), one of the most popular drug/growth factor carriers, have been widely used in diagnostics and biomedicine (Tang et al., 2012; Mamaeva et al., 2013). However, considering the limited drug/growth factor loading percentage and targeting effect, the introduction of bioactive ions, such as Co and Cu ions, in MSN is expected to have sustained treatment effects (Yuan et al., 2012; Xia et al., 2014). Compared with growth factors or drugs, bioactive ions usually induce physiological effects at relatively low concentrations and are less sensitive to pH or temperature under microenvironmental conditions (Li H. et al., 2014). It is evident that bioactive ions (e.g., Co, Cu, and Si) could enhance the osteogenic ability of rabbit mesenchymal stem cells (rBMSCs) and inhibit active bone resorption to increase bone density (Mir et al., 2007). Therefore, it is promising to prepare bioactive ion-containing MSN with uniform morphology and satisfactory degradation performance for application in tissue engineering.

Given the importance and difficulty associated with regenerating large bone defects in tissue engineering, increasing efforts have been made to develop novel bioactive biomaterial-based scaffolds (Kaplan et al., 2020; Matai et al., 2020). The 3D printing method, as a rapid and individual process, has been widely used to fabricate bioactive scaffolds, which can provide a three-dimensional (3D) environment for cell growth and nutrition transfer (Kim et al., 2018; Hassan et al., 2019). Thus, to realize the aim of large bone defect regeneration, it is essential to design functional, bioactive ion-containing MSN-modified 3D-printing bioceramic scaffolds, with interconnected macropore structure, ordered mesostructure, and bioactive ions. To our knowledge, there are only a few bioactive ion-containing MSN-modified 3D-printing bioceramic scaffolds for tissue regeneration. However, reported ion-containing MSN-modified bioceramic scaffolds for bone tissue regeneration do not exhibit the ability of bone tumor therapy.

Recently, owing to its noninvasive and selective characteristics, photothermal therapy has received increasing attention (Chen et al., 2017; Liu et al., 2019; Zhang et al., 2019; Zhou et al., 2020). Compared with carbon-based agents and organic systems (polypyrrole, polyaniline, and polydopamine), Cu-based photothermal agents, such as CuS, Bi_2_S_3_, CuFeSe_2_, and CuCo_2_S_4_, have excellent photothermal performance with the advantages of easy fabrication, tunable size, good photostability, and low cost (Liu and Su, 2014; Zhang et al., 2016; Figueroa et al., 2017; Li et al., 2017). Therefore, copper-based biomaterials have been hypothesized to have great potential for tumor treatment.

On the above basis, Cu-containing MSN-modified β-TCP scaffolds (Cu-MSN-TCP) were designed and prepared by 3D printing and spin coating method, with a hierarchically porous structure and functional surface. The excellent photothermal ability of bifunctional scaffolds were systematically investigated. Bifunctional scaffolds exhibited satisfactory photothermal tumor therapy efficiency *in vitro*. Moreover, the results indicated that bifunctional scaffolds had the effect of promoting the osteogenic capacities of rBMSCs. Therefore, the prepared Cu-containing MSN-modified β-TCP scaffolds have the possibility to treat large bone defects caused by tumor.

## Materials and Methods

### Materials

β-TCP powder was purchased from Kunshan Chinese Technology New Materials Corporation (Kunshan, China). Cetyltrimethylammonium bromide (CTAB), ammonium fluoride (NH_4_F), tetraethoxysilane (TEOS), copper nitrate trihydrate, pluronic F-127, calcein AM, ethidium homodimer-1, fluorescein isothiocyanate (FITC), and 4,6-diamidino-2-phenylindole (DAPI) were purchased from Sigma-Aldrich (Shanghai, China). Cell Counting Kit-8 (CCK-8) was purchased from Beyotime Biotechnology (China).

### Synthesis of Cu-MSN-TCP Scaffolds

First, we used a 3D printing method to fabricate β-TCP scaffolds. Mixed inks (5 g of β-TCP powder and 3.4 g of F127 solution) were stirred to obtain a homogeneous mixture and were subsequently loaded in a printing tube and printed *via* a computer-assisted design model.

Second, 5% Cu containing-MSN (5% Cu-MSN) were synthesized according to a method reported previously (Shi et al., 2016). In brief, CTAB (1.8 g) and NH_4_F (3 g) were dissolved in distilled water (500 ml) and stirred at 80°C for 1 h. Copper nitrate trihydrate [Cu(NO_3_)_2_∙3H_2_O: 0.51 g] was dissolved in ethanol solution (1 ml), which was mixed with TEOS (9 ml), and the mixture was added dropwise to the solution of CTAB (1.8 g) and NH_4_F (3 g). Nanospheres were collected, washed three times, freeze-dried, and calcined for 6 h at 600°C.

Finally, to prepare Cu-MSN-TCP scaffolds, the Cu-MSNs were uniformly coated on β-TCP scaffolds *via* the spin coating method (Zhang et al., 2015). Cu-MSN (0.005 g) was dissolved in 1 ml of dichloromethane:ethanol (20:80 v/v) solution under ultrasonic treatment for 1 h. Then, β-TCP scaffolds were immersed in Cu-MSN solution for 30 s. Thereafter, 20 μl Cu-MSN solution was coated on the surface of bioceramic scaffolds (β-TCP scaffolds) *via* spin coating at 500 rpm for 20 s. This procedure was repeated two, four, six, and eight times to prepare 2Cu-MSN-TCP, 4Cu-MSN-TCP, 6Cu-MSN-TCP, and 8Cu-MSN-TCP scaffolds, respectively.

### Characterization

Microstructures of Cu-MSNs and Cu-MSN-TCP scaffolds were investigated using scanning electron microscopy (SEM, Mira3/MIRA3,TESCAN,Czech) and transmission electron microscopy (TEM, TALOS F200X, FEI, USA). Energy-dispersive spectroscopy (EDS) analysis was performed *via* TEM using an energy-dispersive spectrometer.

To detect the release of Cu, Si, and Ca ions from β-TCP and Cu-MSN-TCP scaffolds, the scaffolds were soaked in phosphate-buffered saline (PBS) for 1, 4, and 7 days (PBS volume to scaffold mass was 200 ml/g). On days 1, 4, and 7, the solution was collected and replaced with fresh PBS solution. The released Cu, Ca, and Si ion concentrations from the collected PBS solution were detected *via* inductively coupled plasma mass spectrometry (ICP-MS, I CAP Q, Thermo Fisher, Germany) and inductively coupled plasma optical spectrometry (iCAP 6300, Thermo, USA).

### Photothermal Performance Test

To investigate their photothermal ability, 2Cu-MSN-TCP, 4Cu-MSN-TCP, 6Cu-MSN-TCP, and 8Cu-MSN-TCP scaffolds were irradiated for 500 s using a near-infrared (NIR) laser (power density: 0.9 W/cm^2^). In addition, the effect of different laser power densities on the final temperature of scaffolds was assessed, and the temperature was recorded using an IR thermal camera (Flir SC325, FLIR Systems Inc., USA).

### *In vitro* Antitumor Efficacy

MG-63 cells (5.0 × 10^4^), cultured in Dulbecco's modified Eagle's medium, were seeded in each well of a 48-well plate for 24 h (37°C, 5% CO_2_) in an incubator and received the following four treatments: β-TCP scaffold without irradiation, β-TCP scaffold with radiation, 8Cu-MSN-TCP scaffold without irradiation, and 8Cu-MSN-TCP scaffold with irradiation. Three scaffolds in each group were used for study. The scaffolds were gently placed into wells and irradiated using NIR laser (time: 15 min, power density: 2.5 W/cm^2^) to investigate the antitumor efficacy of bifunctional scaffolds against the surrounding tumor cells. Subsequently, the scaffolds were removed from the medium. Tumor cell viability was detected after 12 h using the CCK 8 assay according to the manufacturer's protocol. To obtain a more satisfactory antitumor efficacy, scaffolds were irradiated twice.

To visually observe the antitumor efficacy of bifunctional scaffolds following NIR light irradiation, calcein AM and ethidium homodimer-1 solutions were used for Live/Dead staining of tumor cells. Cells were observed using a confocal laser scanning microscope (CLSM; Leica TCS SP8, Wetzlar, Germany). Live tumor cells showed green fluorescence, whereas dead tumor cells showed red fluorescence.

### *In vitro* Biocompatibility and Osteogenic Differentiation Assay

The third passage of rabbit bone marrow-derived mesenchymal stem cells (rBMSCs, 3.0 × 10^4^) was cultured on scaffolds (β-TCP and 8Cu-MSN-TCP) placed in 24-well plates containing the Minimum Essential medium for 1, 3, and 5 days. Three scaffolds in each group were used for study. On days 1, 3, and 5, cell viability was investigated by the CCK-8 assay and the absorbance measured using a microplate reader (Epoch, BIO-TEK, USA).

To observe cell attachment on scaffolds, 5.0 × 10^4^ rBMSCs were cultured on scaffolds (β-TCP, 8Cu-MSN-TCP). Five days later, scaffolds were washed three times with PBS and subsequently treated with 2.5% glutaraldehyde for 20 min. Thereafter, samples were dehydrated using a gradient series of ethanol solutions (30, 50, 70, 80, 90, 95, and 100% v/v). Cell morphology on the scaffolds was observed *via* SEM.

Cell distribution and the cytoskeleton were characterized using a CLSM. After 5.0 × 10^4^ rBMSCs were cultured on each scaffold (β-TCP, 8Cu-MSN-TCP) for 5 days, the scaffolds were treated with 2.5% glutaraldehyde. The cytoskeleton and nuclei of rBMSCs on scaffolds were stained with FITC solution for 1 h and DAPI solution for 10 min, respectively. Thereafter, the stained cells were observed by fluorescence microscopy (TCS SP8, Leica, Germany).

To investigate the effect of 8Cu-MSN-TCP scaffolds on the osteogenic differentiation of rBMSCs, 2.0 × 10^5^ rBMSCs were cultured on each scaffold (β-TCP, 8Cu-MSN-TCP) in six-well plates for 7 days. Three scaffolds in each group were used for study. Real-time quantitative reverse transcription polymerase chain reaction (RT-qPCR) was conducted using SYBR Green qPCR Master Mix (TaKaRa, Japan) with a Light Cycler apparatus (CFX-Touch, Bio-Rad, USA) according to a previously reported method (Lin et al., 2019). The primers used in PCR are listed in Table 1.

**Table 1:** Primer sequences used in quantitative reverse-transcriptase polymerase chain reaction.

**Gene**	**Primer sequences**
Rabbit-VEGF-F	**5′-**CGCTGGATTTCCATACTCAT**-3′**
Rabbit-VEGF-R	**5′-**TCTGCCCGTCTTCAGTG**-3′**
Rabbit-OPN-F	**5′-**TCTCAGAAGCAGAATCTCCTAAC**-3′**
Rabbit-OPN-R	**5′-**ATGGCTTTCAATGGACTTACTC**-3′**
Rabbit-Runx2-F	**5′-**CCTTCCACTCTCAGTAAGAAGA**-3′**
Rabbit-Runx2-R	**5′-**TAAGTAAAGGTGGCTGGATAGT**-3′**
Rabbit-BSP-F	**5′-**GAGCCTATGAGGATGAGTACAGTTA**-3′**
Rabbit-BSP-R	**5′-**AAATGGTCGCCAAATGC**-3′**
Rabbit-BMP-2-F	**5′-**GGCCTTTGCTCGTAACTT**-3′**
Rabbit-BMP-2-R	**5′-**TCCGCTGTTTGTGTTTCG**-3′**
Rabbit-GAPDH-F	**5′-**GACTTCAACAGTGCCACC**-3′**
Rabbit-GAPDH-R	**5′-**TGCTGTAGCCAAATTCGT**-3′**

## Results and Discussion

### Fabrication and Characterization of Scaffolds

**Figure 1:**
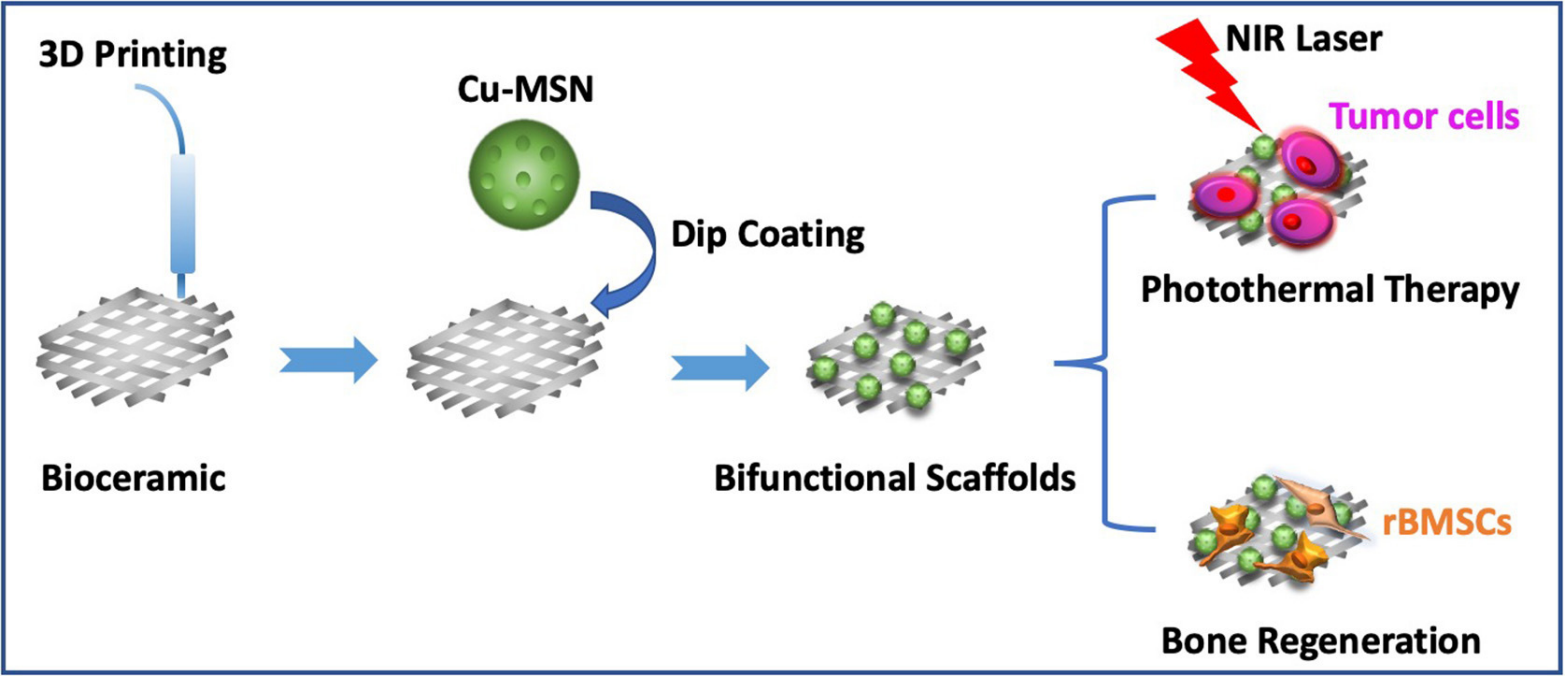
Schematic illustration of prepared Cu-containing mesoporous silica nanospheres modified β-TCP scaffolds by combining 3D printing and spin coating method, and potential application of prepared scaffolds in photothermal tumor therapy and simultaneous osteogenesis promotion.

**Figure 2:**
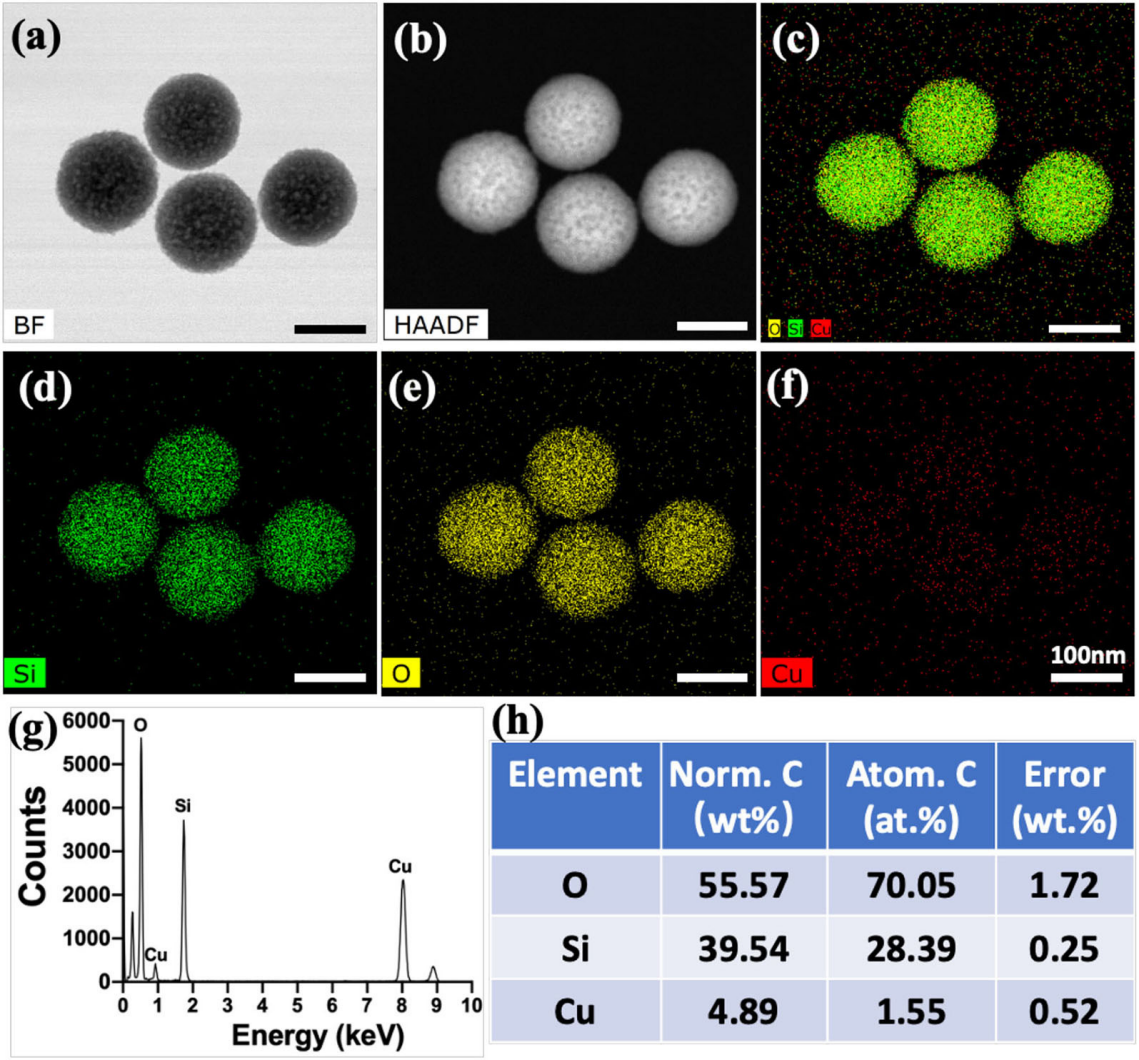
Transmission electron microscope images of Cu-containing mesoporous silica nanospheres (Cu-MSN) in bright field (BF) model **(a)** and high-angle annular dark field (HAAD) model **(b)**. Energy-dispersive spectrometry element mapping images of all **(c)**, Si **(d)**, O **(e)**, and Cu **(f)** elements distributed in Cu-MSN. Energy-dispersive spectroscopy analysis **(g,h)** of Cu-MSN.

In this study, Cu-MSN-TCP scaffolds were fabricated *via* 3D printing and spin coating for the application of bone tumor therapy and tissue regeneration (Figure 1). First, 5% Cu-MSN were prepared using a soft-template method (CTAB). TEM and SEM results showed that all Cu-MSN exhibited a uniform spherical morphology with a well-ordered mesostructure, and the average diameter of Cu-MSN was approximately in a range of 120–145 nm (Figures 2a,b and Supplementary Figure 1). According to the element mapping images obtained from EDS, uniform distribution of Si, O, and Cu elements in Cu-MSN was observed (Figures 2c–f). EDS verified the existence of Cu, and the mass fraction of Cu accounted for 4.890.52% (Figures 2g,h). The above results suggest successful synthesis of 5% Cu-MSN with an ideal structure, which is attributed to the cooperative assembly pattern between Cu and the structure-directing agent (CTAB), leading to a homogeneous distribution of Cu (Wei et al., 2009).

**Figure 3:**
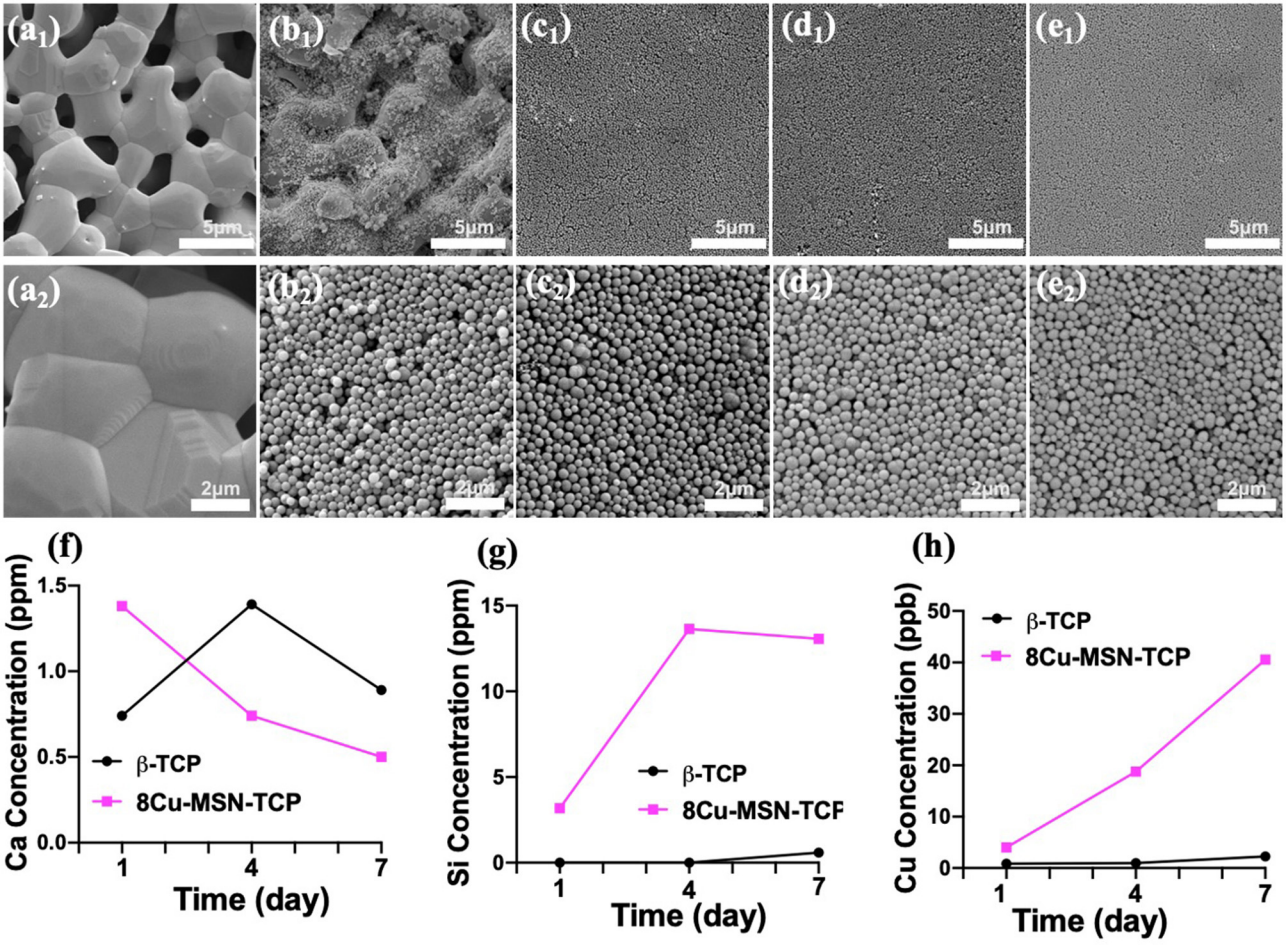
Scanning electron microscope images of β-tricalcium phosphate (β-TCP) **(a**_**1**_**,a**_**2**_**)**, 2Cu-MSN-TCP **(b**_**1**_**,b**_**2**_**)**, 4Cu-MSN-TCP **(c**_**1**_**,c**_**2**_**)**, 6Cu-MSN-TCP **(d**_**1**_**,d**_**2**_**)**, and 8Cu-MSN-TCP **(e**_**1**_**,e**_**2**_**)** scaffolds. The concentration of released Ca **(f)**, Si **(g)**, and Cu **(h)** ions from β-TCP and 8Cu-MSN-TCP scaffolds in phosphate-buffered saline (PBS) on days 1, 4, and 7.

Thereafter, to prepare Cu-MSN-TCP scaffolds, the Cu-MSN solution was coated onto the surface of 3D-printed β-TCP scaffolds *via* the spin coating method. SEM analysis was conducted to assess the effect of spin coating times on the microstructure of Cu-MSN-TCP scaffolds. Low-magnification SEM images showed that after spin coating four, six, or eight times, Cu-MSN-TCP scaffolds were denser than those after two cycles (Figures 3a_1_–e_1_). As shown in Figures 3a_2_–e_2_, after spin coating four, six, or eight times, the nanolayers of Cu-MSN were uniformly deposited on the surface of β-TCP scaffolds. Herein, a combined method of 3D printing and spin coating was used to fabricate bioceramic scaffolds with hierarchical structures, including large pores and ordered mesostructures. Such an assembly strategy is extremely facile and effective.

### Ion Release From Cu-MSN-TCP Scaffolds

The release of Ca, Si, and Cu ions in PBS solution was detected *via* ICP-AES. As shown in Figures 3f–h, there was an evident continuous release of Si and Cu in 8Cu-MSN-TCP scaffolds. The concentration of Cu ions released from 8Cu-MSN-TCP scaffolds was approximately 4.01, 18.79, and 40.56 ppb on days 1, 4, and 7, respectively. The concentration of released Si ions from Cu-MSN-TCP scaffolds was 3.18, 13.64, and 13.07 ppm on days 1, 4, and 7, respectively. As a control, a negligible amount of released Cu and Si ions were detected from β-TCP scaffolds on days 1, 4, and 7. The continuous release of Si and Cu ions may favor osteogenesis during bone defect regeneration (Wu et al., 2019).

### Photothermal Performance of Cu-MSN-TCP Scaffolds

**Figure 4:**
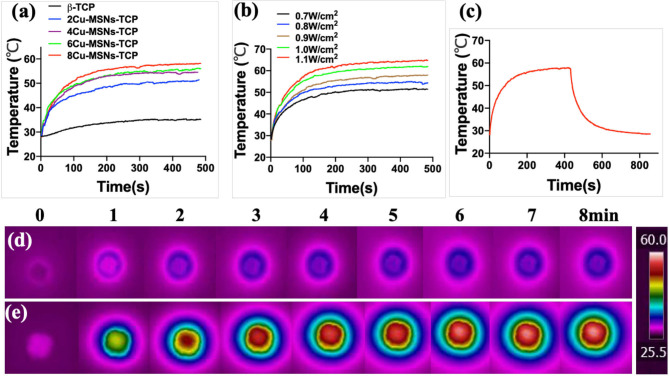
Cu-containing mesoporous silica nanospheres modified β-tricalcium phosphate **(**Cu-MSN-TCP) scaffolds exhibited a coating time-dependent **(a)** and laser power density-dependent **(b)** photothermal performance. The photothermal heating and cooling curve of 8Cu-MSN-TCP scaffolds under irradiation at 0.9 W/cm^2^ **(c)**. Visual thermal images and temperature change of β-TCP **(d)** and 8Cu-MSN-TCP **(e)** scaffolds can be recorded and obtained using an infrared (IR) thermal camera.

Cu-MSN-TCP scaffolds exhibited coating time- and laser power density-dependent photothermal performance. The temperature of 8Cu-MSN-TCP scaffolds rapidly reached 58°C following NIR laser irradiation for 500 s (power density: 0.9 W/cm^2^), whereas the temperature of the β-TCP scaffold only increased by 7°C following NIR laser irradiation for 500 s at the same power density (0.9 W/cm^2^) (Figure 4a). In addition, the temperature of 8Cu-MSN-TCP scaffolds increased with an increase in NIR laser power density (Figure 4b). The temperature of 8Cu-MSN-TCP scaffolds exhibited a rapid increasing trend with NIR laser irradiation (Figure 4c). Visual thermal images and temperature change were recorded *via* an IR thermal camera, as shown in Figures 4d,e. The excellent photothermal ability of Cu-MSN-TCP scaffolds may be attributed to the d-d electronic transition of Cu ions, leading to the strong absorbance of the NIR light (Tian et al., 2011a,b; Li et al., 2017; Lu et al., 2018). These results indicated that Cu-MSN-TCP scaffolds can act as effective photothermal agents for killing tumor cells.

### Photothermal Therapy Efficiency

**Figure 5:**
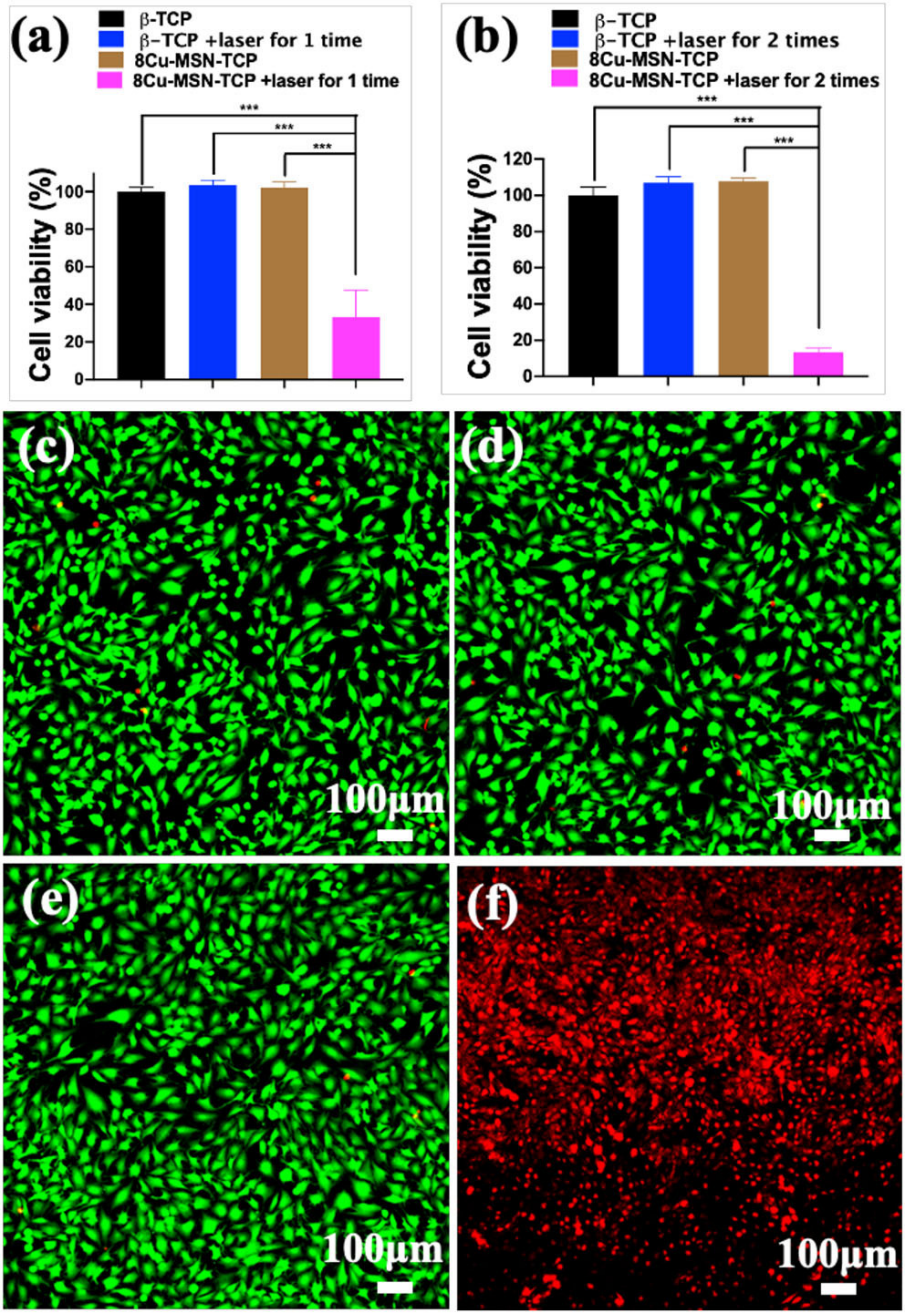
The viability of MG-63 tumor cells, treated with 8Cu-MSN-TCP scaffolds after being irradiated once **(a)** and twice **(b)** (*n* = 3). Confocal laser scanning microscope images of MG 63 tumor cells treated with β-TCP without irradiation **(c)**, β-TCP scaffold with irradiation **(d)**, 8Cu-MSN-TCP without irradiation **(e)**, and 8Cu-MSN-TCP with irradiation **(f)**. MG 63 tumor cells were stained with calcein AM (green fluorescence, live cells) and ethidium homodimer-1 (red fluorescence, dead cells). Hyperthermia induced by 8Cu-MSs-TCP scaffolds had sufficient killing effect on tumor cells.

To assess the efficiency of the photothermal therapy using 8Cu-MSN-TCP scaffolds, CCK 8 analysis was conducted. It was found that compared with the other three groups, the average viability of MG-63 treated with the 8Cu-MSN-TCP scaffolds was 33.12% after being irradiated once (Figure 5a). This further decreased to 13.44% after second irradiation (Figure 5b), suggesting that hyperthermia induced by 8Cu-MSN-TCP scaffolds had a satisfactory killed tumor cells. CLSM images also showed that almost all tumor cells in the other three groups were viable (green fluorescence; Figures 5c–e). In contrast, a large amount of MG 63 tumor cells were dead (red fluorescence) in the 8Cu-MSN-TCP scaffolds + irradiation group (Figure 5f). Thus, Cu-MSN-TCP scaffolds are excellent photothermal agents, which exhibited effective photothermal therapy *in vitro* under NIR laser irradiation, as hyperthermia (>50°C) can cause cell membrane damage, cause irreversible protein denaturation, and induce cell apoptosis and necrosis (Chu and Dupuy, 2014; Ma et al., 2016; Yin et al., 2016).

### *In vitro* Biocompatibility of rBMSCs

**Figure 6:**
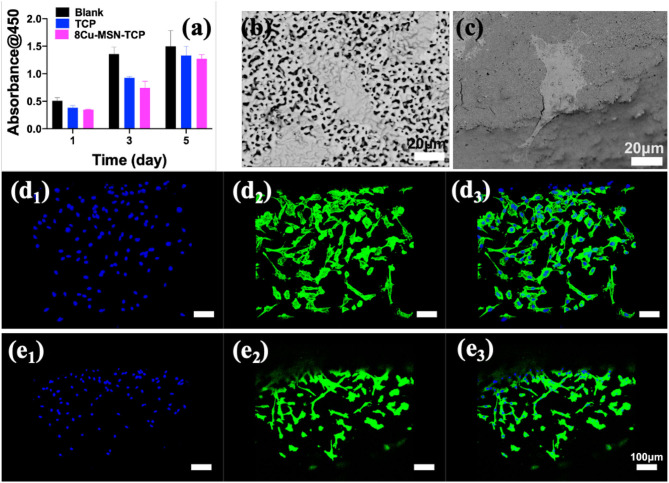
The viability of rabbit bone marrow-derived mesenchymal stem cells in β-TCP and 8Cu-MSN-TCP scaffolds for 1, 3, and 5 days **(a)** (*n* = 3). Scanning electron microscopy **(b,c)** and confocal microscopy images of nucleus **(d**_**1**_**,e**_**1**_**)**, cytoskeleton **(d**_**2**_**,e**_**2**_**)** and merged mode **(d**_**3**_**,e**_**3**_**)** of rBMSCs of rBMSCs in β-TCP **(b,d)** and 8Cu-MSN-TCP scaffolds **(c,e)**, suggesting that 8Cu-MSN-TCP scaffolds had satisfactory biocompatibility.

It is necessary to explore not only the photothermal therapeutic effect of bifunctional scaffolds but also their biological activity. The viability of rBMSCs in β-TCP and 8Cu-MSN-TCP scaffolds at 1, 3, and 5 days is shown in Figure 6a. A cell proliferation increasing trend with culture time was evident in both β-TCP and 8Cu-MSN-TCP scaffold groups. Meanwhile, SEM (Figures 6b,c) and confocal microscopy (Figures 6d_1_–d_3_,e_1_–e_3_) were used to observe the morphology and attachment of rBMSCs on scaffolds on day 3. The 8Cu-MSN-TCP scaffolds supported cell adhesion and distribution, and rBMSCs were in close contact with the surface of Cu-MSN (Supplementary Figure 2). In addition, stained cells on 8Cu-MSN-TCP scaffolds exhibited a well-defined cytoskeleton as well as evident filopodia. Collectively, these results suggested that the Cu-MSN-TCP scaffolds had satisfactory biocompatibility.

### *In vitro* Osteogenic Differentiation Assay

**Figure 7:**
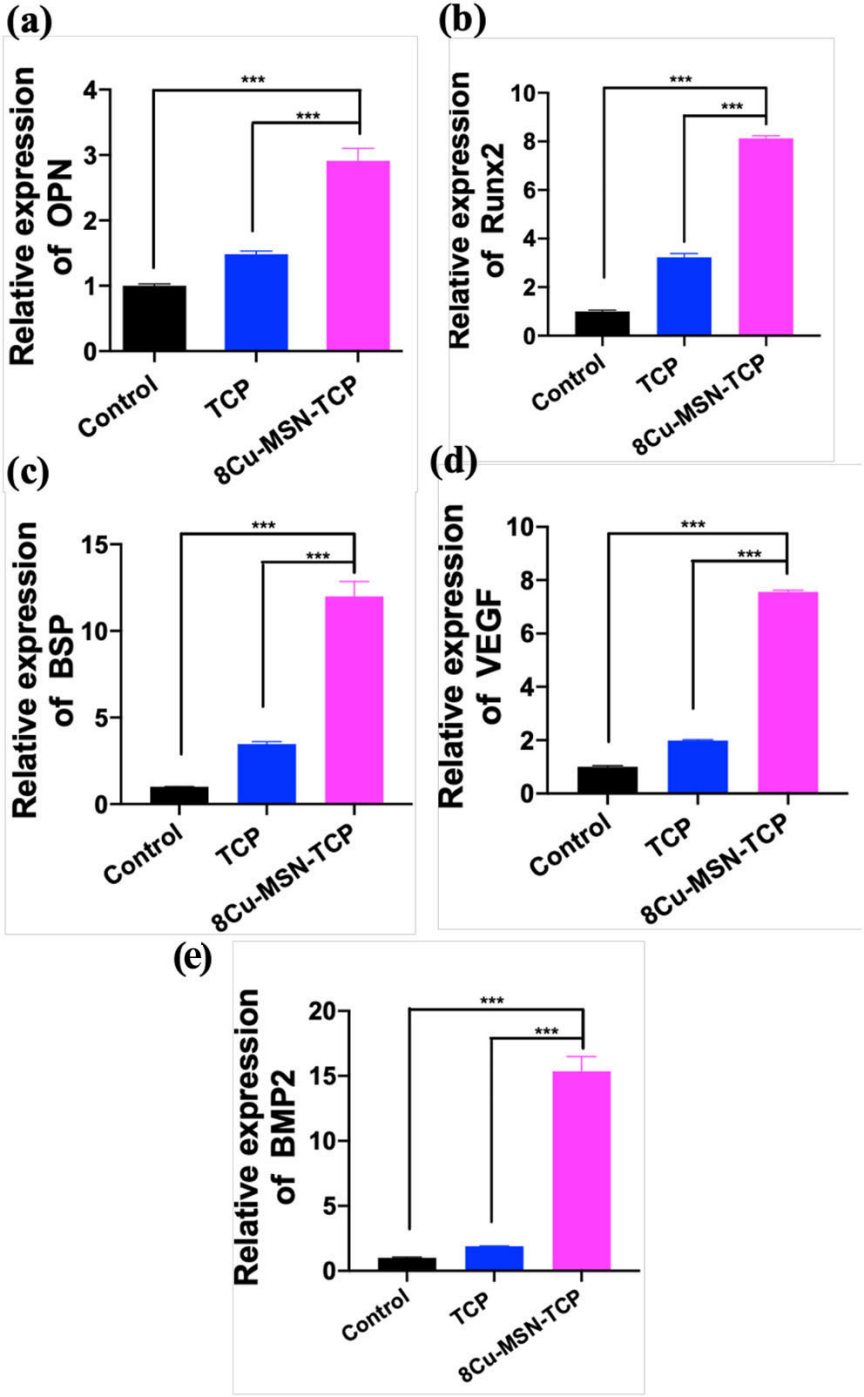
The osteogenic differentiation of rabbit bone marrow-derived mesenchymal stem cells was assessed by RT-qPCR. The gene expression of OPN **(a)**, Runx2 **(b)**, BSP **(c)**, VEGF **(d)**, BMP 2 **(e)** in the blank group, β-TCP, and 8Cu-MSN-TCP scaffolds (*n* = 3). 8Cu-MSN-TCP scaffolds can recognize the promotion of osteogenesis.

RT-qPCR was used to evaluate the osteogenic ability of rBMSCs in β-TCP and 8Cu-MSN-TCP scaffolds. Gene expression of osteogenic markers, OPN, Runx2, BSP, BMP2, VEGF, on 8Cu-MSN-TCP scaffolds was significantly higher than that on blank and β-TCP scaffolds, suggesting that 8Cu-MSN-TCP scaffolds can promote osteogenesis (Figures 7a–e). It has been reported that the constant release of Si and Ca ions from 8Cu-MSN-TCP scaffolds favors collagen synthesis, metabolism, and bone mineralization and increases the osteogenic differentiation of BMSCs and osteoblasts (Valerio et al., 2004; Wang et al., 2013; Li X. et al., 2014). Furthermore, Cu ions can modulate the immune environment, induce osteogenic factors, and suppress osteoclastogenic factors through immune cells (Mroczek-Sosnowska et al., 2015; Shi et al., 2016).

A key challenge in bone tissue engineering is the design and fabrication of bifunctional scaffolds for both therapy and regeneration. To our knowledge, there are only a few MSN-modified 3D-printing bioceramic scaffolds for bone tissue regeneration. However, reported ion-containing MSN-modified bioceramic scaffolds for bone tissue regeneration do not exhibit the ability of bone tumor therapy. One of important results was that Cu-MSN-TCP scaffolds exhibited excellent photothermal performance, which can function as photothermal agents for killing tumor cells. Therefore, the novelty of this work is the fabrication of novel bifunctional scaffolds for both tumor therapy and tissue engineering.

There are several limitations to our study. First, because of excellent photothermal property of Cu-MSN-TCP scaffolds, they can kill tumor cells effectively under NIR laser irradiation. It would be worth investigating the effect of tumor therapy and bone regeneration of Cu-MSN-TCP scaffolds *in vivo*. In addition, considering the penetration depth of NIR laser, these bifunctional scaffolds also face some details and problems in clinical applications. Finally, Cu-MSN-TCP scaffolds can function as carriers of anti-cancer drugs or immune checkpoint inhibitors, and future work can focus on synergistic therapy of bone tumor.

## Conclusion

A facile method of combining 3D printing and spin coating was applied to develop Cu-MSN-TCP scaffolds. Prepared 8Cu-MSN-TCP scaffolds had a dense uniform nanolayer of spherical morphology with a well-ordered mesostructure. Notably, these scaffolds exhibited excellent photothermal activity and could effectively kill tumor cells, owing to controlled hyperthermia induced under NIR laser irradiation. Furthermore, the scaffolds maintained satisfactory biocompatibility and evidently promoted gene expression of osteogenic markers OPN, Runx2, BSP, BMP 2, and VEGF. Therefore, this work indicates that the prepared Cu-MSN-TCP scaffolds can act as potential bifunctional biomaterials for photothermal bone cancer therapy and simultaneous bone defect regeneration. In the future, such Cu-MSN-TCP scaffolds may function as carriers of anti-cancer drugs or immune checkpoint inhibitors in chemo-/photothermal or immune-/photothermal therapy of bone tumors, favoring for effective treatment.

## Data Availability Statement

The datasets presented in this study can be found in online repositories. The names of the repository/repositories and accession number(s) can be found in the article/Supplementary Material.

## Author Contributions

JW and HuM contributed to study conception and design. HoM and ZM initiated and conducted this study. QC, WL, ZM, and HoM contributed to data collection, assembly, and analysis. HoM visualized data. XL, XM, and HY contributed to the literature review. HoM and ZM conceived this article and wrote the first draft. YM, HuM, and JW critically revised the article. All authors contributed to the article and approved the submitted version.

## Conflict of Interest

The authors declare that the research was conducted in the absence of any commercial or financial relationships that could be construed as a potential conflict of interest.
