# Supplementary material for: Bifunctional, Copper-Doped, Mesoporous Silica Nanosphere-Modified, Bioceramic Scaffolds for Bone Tumor Therapy
Source: Front Chem. 2020 Dec 9;8:610232. doi: 10.3389/fchem.2020.610232 (PMC7755992; doi:10.3389/fchem.2020.610232)
Supplement: Supplementary file 1 [file Data_Sheet_1.docx]

**Supporting Information**

Bifunctional, copper-doped, mesoporous silica nanosphere-modified, bioceramic scaffolds for bone tumor therapy

**Hongshi Ma^1#^, Zhenjiang Ma ^1#^, Qufei Chen^1^, Wentao Li^1^, Xiangfei Liu^2^,** **Xiaojun Ma^3^,** **Yuanqing Mao^1^, Han Yang^1^, Hui Ma^1*^, Jinwu Wang^1*^**

^1^Shanghai Key Laboratory of Orthopaedic Implants, Department of Orthopaedic Surgery, Shanghai Ninth People’s Hospital, Shanghai Jiao Tong University School of Medicine, Shanghai 200011, P. R. of China.

^2^Shanghai Zhongye Hospital, Department of Orthopaedic Surgery, Shanghai 201900, P. R. of China.

^3^Department of Orthopedics, Shanghai General Hospital, Shanghai Jiao Tong University School of Medicine, Shanghai 200080, China.

# Hongshi Ma and Zhenjiang Ma share the co-first author.

***Correspondence:***Corresponding author: Jinwu Wang, Hui Ma. Jinwu Wang will handle correspondence at all stages of refereeing and publication, as well as post-publication

Email: wangjw@shsmu.edu.cn (Jinwu Wang), Orthopain@163.com (Hui Ma)

**
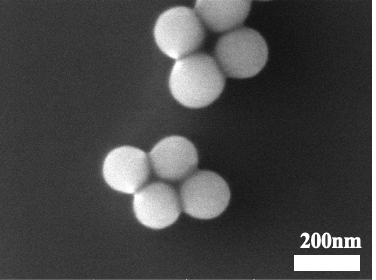
**

**Figure S1.** Scanning Electron Microscope images of Cu-containing mesoporous silica nanospheres.

**
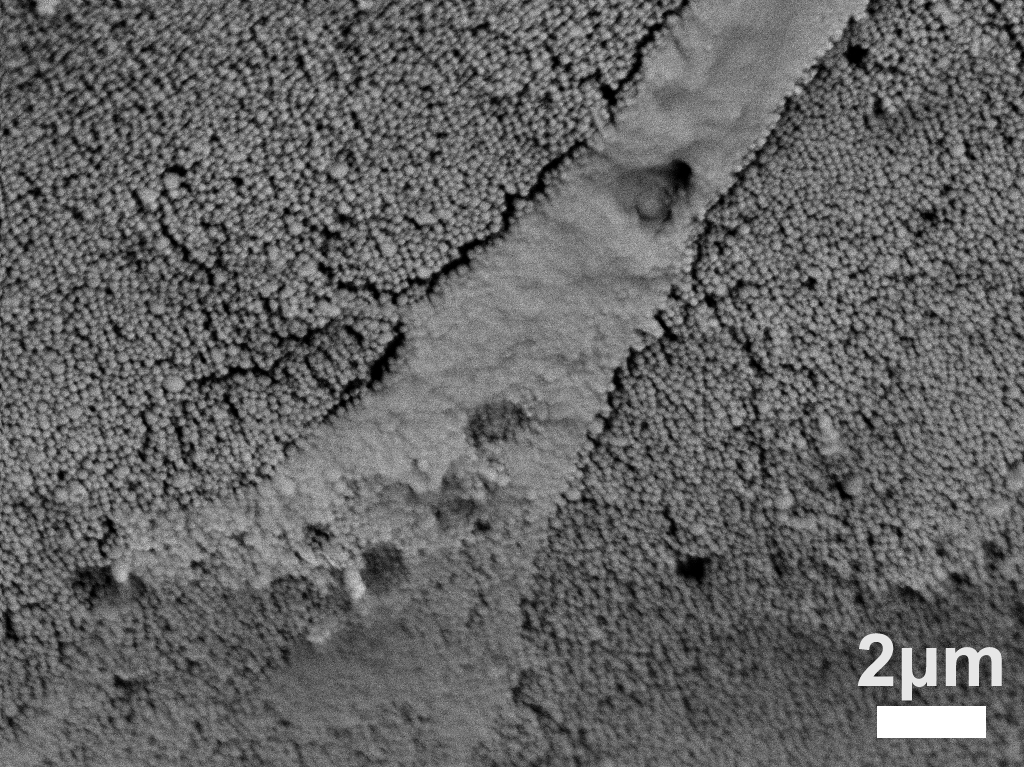
**

**Figure S2.** Scanning Electron Microscope images of rBMSCs on 8Cu-MSN-TCP scaffolds.
